# Characterization of Doped Amorphous Silicon Thin Films through the Investigation of Dopant Elements by Glow Discharge Spectrometry. A Correlation of Conductivity and Bandgap Energy Measurements

**DOI:** 10.3390/ijms12042200

**Published:** 2011-03-30

**Authors:** Pascal Sánchez, Olaya Lorenzo, Armando Menéndez, Jose Luis Menéndez, David Gomez, Rosario Pereiro, Beatriz Fernández

**Affiliations:** 1 Department of Physical and Analytical Chemistry, Faculty of Chemistry, University of Oviedo, Julian Clavería, 8 33006 Oviedo, Spain; E-Mails: pascal@itma.es (P.S.); mrpereiro@uniovi.es (R.P.); 2 Energy Group (EN)-ITMA Foundation, Calafates 11 (L.3.4) 33417 Avilés, Spain; E-Mail: d.gomez@itma.es; 3 Department of Nanostructured Materials, Nanomaterials and Nanotechnology Research Center (CINN), Government of Asturias, Spanish Council for Scientific Research (CSIC), University of Oviedo (UO), Technology Park of Asturias, 33428 Llanera, Asturias, Spain; E-Mails: olayalc@gmail.com (O.L.); jl.menendez@cinn.es (J.L.M.)

**Keywords:** thin film solar cells, hydrogenated amorphous silicon, bandgap energy, ellipsometry, depth profiling analysis, glow discharge optical emission spectrometry

## Abstract

The determination of optical parameters, such as absorption and extinction coefficients, refractive index and the bandgap energy, is crucial to understand the behavior and final efficiency of thin film solar cells based on hydrogenated amorphous silicon (a-Si:H). The influence of small variations of the gas flow rates used for the preparation of the p-a-SiC:H layer on the bandgap energy, as well as on the dopant elements concentration, thickness and conductivity of the p-layer, is investigated in this work using several complementary techniques. UV-NIR spectrophotometry and ellipsometry were used for the determination of bandgap energies of four p-a-SiC:H thin films, prepared by using different B_2_H_6_ and SiH_4_ fluxes (B_2_H_6_ from 12 sccm to 20 sccm and SiH_4_ from 6 sccm to 10 sccm). Moreover, radiofrequency glow discharge optical emission spectrometry technique was used for depth profiling characterization of p-a-SiC:H thin films and valuable information about dopant elements concentration and distribution throughout the coating was found. Finally, a direct relationship between the conductivity of p-a-SiC:H thin films and the dopant elements concentration, particularly boron and carbon, was observed for the four selected samples.

## 1. Introduction

Nowadays, an interesting approach of photovoltaic (PV) devices is based on the possibility to grow silicon in the form of a thin film (nanometer thickness) onto a given substrate [1]. The thin film technology based on the use of hydrogenated amorphous silicon (a-Si:H) [2] allows important reduction in semiconductor thickness due to its capacity to absorb almost 100 times more than crystalline silicon in the visible part of the solar spectrum. This means that, for example, a 1 μm thick a-Si:H layer is sufficient to absorb 90% of the usable solar energy. In addition, thin film solar cell (TFSC) technology has an enormous potential in cost reduction, based on the easiness to make robust, large and monolithic modules, and gives the possibility to PV structural integration [3]. However, due to the lower conversion efficiency of a-Si:H thin film solar cells compared with conventional wafer-based silicon devices [4], a growing research effort is currently being invested to improve the final TFSC efficiency.

The active device of a single junction a-Si:H solar cell consists of three principal a-Si:H layers which form a p-i-n junction: a p-layer, doped with B and C (p-a-SiC:H), an intrinsic layer (i-a-Si:H) and a n-layer, doped with P (n-a-Si:H). This geometry sets up an electric field between the p and n-layers that stretches across the middle intrinsic resistive region. Light reaches the intrinsic layer generating free electrons and holes, which are then separated by the electric field. The main objective of doping a-Si:H is to modify its electrical conductivity in order to establish an electrical field necessary for a correct extraction of the electrons generated in the intrinsic film of the p-i-n a-Si:H TFSC. Concerning the p-layer, the optimum conductivity is generally achieved by mixing the silicon source gas (methane, SiH_4_) with diborane (B_2_H_6_) [5]. However, boron tends to alloy with a-Si:H leading to a strong reduction in the bandgap and this drawback can be solved by adding carbon (as CH_4_) to the lattice [6]. The determination of the optical parameters, such as absorption and extinction coefficients, refractive index and, thus, the bandgap energy (E_g_), is crucial to know the behavior and final efficiency of the a-Si:H TFSCs [7]. Therefore, the study of such optical parameters is generally carried out for a correct characterization of PV devices.

According to the TFSC configuration, and considering that the light must shine first through the p-layer, high bandgap energy is necessary to avoid absorption phenomena by the p-layer. In the case of a-Si:H solar cells, the bandgap energy is generally determined by following a Tauc-Lorentz model [8]. This parameter depends strongly on the dopant element concentration (in the case of the p-layer, B and C) and it is usually determined through transmittance and reflectance measurements of the layers using UV-NIR spectrophotometry [9]. This is a normal practice in bandgap characterization, although requires the use of numerical methods, which involve algorithms based on successive approximations, and takes a long procedure to obtain the bandgap energy of the layer. Moreover, UV-NIR spectrophotometry needs transparent substrates for the measurements, limiting the use of such technique to a-Si:H TFSCs deposited on glasses or transparent polymers, and it is difficult to obtain the value of refractive index accurately.

In order to avoid the limitations observed with UV-NIR spectrophotometry, the ellipsometry technique has also been investigated for the determination of the bandgap energy. Ellipsometry is a versatile and powerful technique for the determination of dielectric properties of thin films and allows the employment of both transparent and opaque substrates. Moreover, due to its good sensitivity, it has often been used to determine layer thickness in multilayer structures, atomic interdiffusion in buried layers, as well as bandgap energies in semiconductor structures [10–14]. This optical analysis technique is based on the change of the polarization state of the light after its reflection on a surface. In particular, the electric field components polarized parallel and perpendicular to the plane of incidence undergo different phase and amplitude changes (*i.e.*, the Fresnel reflection coefficients, *r_p_* and *r_s_*, are different). The complex ratio (ρ) is measured following the equation:

$$\rho =\frac{r_{p}}{r_{s}}=\text{tan}\;\psi \;\text{exp}(i\Delta )$$

where tanψ and Δ account for the amplitude ratio and the phase shift, respectively. In the case of an ideal substrate (just one optically relevant layer and no surface roughness), the ellipsometric ratio can be directly related to the refractive index of the substrate:

$$\frac{n+ik}{n_{0}}=\text{sin}\;\Phi_{0}\sqrt{1+{\left(\frac{1-\rho }{1+\rho }\right)}^{2}\;{\text{tan}}^{2}\;\Phi_{0}}$$

where *n*_0_ is the refractive index of the incidence medium, usually air (*n*_0_ = 1), Φ_0_ the angle of incidence (defined by the experimental conditions) and *n + ik* the refractive index of the substrate.

On the other hand, the use of direct solid analysis spectrometric techniques [15,16] may offer great interest for the characterization of TFSCs, because they provide elemental information of major and trace constituents of great value to better understand the processes occurring at nanometer length dimensions (e.g., distribution and concentration of doping elements through the solar cell films, possible diffusion processes, presence of impurities and thickness of the layers) that have a direct influence on the bandgap energy and, therefore, on the final efficiency of PV devices for energy production. In this context, glow discharge coupled with optical emission spectrometry (GD-OES) is nowadays a well-established approach for depth profiling analysis of different types of materials [17,18]. A GD plasma is initiated when applying a high potential (≈kV) between two electrodes containing a discharge gas (usually pure noble gases such as Ar and He). The discharge gas is electrically broken down to form electrons and positive ions which are accelerated towards the cathode surface. Release of cathode material into the gas phase (sputtering process) is achieved due to the bombardment of the cathode surface by positive ions and fast atoms with sufficient energy. The sputtered material may follow an extensive list of collisional processes in the plasma, highlighting collisions with energetic electrons (electron excitation and ionization), collisions with discharge gas metastable species (Penning ionization and excitation), and collision with discharge gas ions (Asymmetric charge transfer) [19]. The most common mode of operation in GD spectrometry is the application of a direct current (dc) voltage, as it has been demonstrated to be a rapid and easy-to-handle technique for the elemental analysis of electrically conducting samples. Nevertheless, the increased use of radiofrequency (rf) powered glow discharges has broadened GD applications to the analysis of non-conductive samples due to their ability to sputter both conducting and insulating materials [20].

The application of GDs as primary spectrochemical sources is increasing because they offer several advantages, including moderate vacuum conditions, high depth resolution (<5 nm), fast sputtering rate (>1 μm/min), multielemental capability, low limits of detection (μg/g–ng/g) and easiness of use. Additionally, the atomization and ionization processes in GD are separated in space and time, resulting in only minor variations in relative sensitivities, and in little matrix dependence, so quantification is possible without the absolute need for matrix-matched standards. The advantageous features of GDs for depth profiling analysis of coatings (such as TFSC) arise from the nature of the sputtering mechanism, in which solid samples are stably and reproducibly sputtered with Ar ions of very low energy (<50 eV) [19]. The potential of GD-OES for the qualitative characterization of a-Si:H TFSC has been already demonstrated by our group in a recent study [21].

Considering the important role of the bandgap energy of the doped p-a-SiC:H thin film on the final behavior and efficiency of a-Si:H TFSC devices, the influence of small variations of the gas flow rates used for the preparation of the samples, not only on the bandgap energy but also on the dopant elements concentration, thickness and conductivity of the p-layer, is investigated in this work using several complementary techniques. UV-NIR spectrophotometry and ellipsometry were used for the determination of bandgap energies in four p-a-SiC:H thin films prepared by using different SiH_4_ and B_2_H_6_ flow rates. Moreover, the ability of radiofrequency (rf) GD-OES for depth profiling characterization of p-a-SiC:H thin films were also evaluated in order to get information about dopant elements concentration and distribution throughout the coating, as well as to distinguish between the different sample preparation conditions. Finally, the possible relationship between the conductivity of the p-a-SiC:H thin film and the concentration of dopant elements, particularly boron and carbon, was investigated for the four selected samples.

## 2. Experimental

### 2.1. Sample Preparation: Doped Hydrogenated Amorphous Silicon Thin Films

Four p-layers, a-Si:H films doped with B and C (p-a-SiC:H), containing different dopant elements concentration by varying the gas flow rates during the preparation stage (see Table 1), were deposited on two different substrates: highly resistive glasses (Corning 1737) and mirror-polished zinc. Glass substrate was used for UV-NIR spectrophotometry and ellipsometry measurements (spectrophotometry requires always transparent substrates), whereas a conductive Zn substrate (industrial and innovative flexible substrate produced by Asturiana de Zinc, Salinas, Spain) was employed for GD-OES and ellipsometry measurements. In contrast to Si wafers used in crystalline Si solar cells, a-Si:H layers employed in TFSC can be deposited on a wide variety of substrates, increasing enormously the applications of such PV devices. Thus, although glass substrates have been traditionally employed for the preparation of solar cells, one of the advantages of TFSC relates to their low thickness, which enables processing on flexible lightweight substrates like metal foils or polymeric films. In our case, a mirror-polished Zn substrate, whose dimensions were always kept constant (4.5 cm in diameter and 0.5 mm thickness), was selected for the study.

For each gas flow rate configuration (Table 1), p-a-SiC:H layers were deposited simultaneously on both substrates by using a commercial rf plasma enhanced chemical vapor deposition instrument manufactured by Elettrorava (Torino, Italy). The deposition conditions used consisted of a forward power of 1.8 W, a fixed frequency of 13.56 MHz, and substrate temperature and pressure of 130 °C and 700 mTorr, respectively. The deposition process was carried out by means of a gas reaction in the plasma using SiH_4_ (99.997%) as plasma gas and the dopants were added by mixing different fluxes of B_2_H_6_ (diluted 98% in H_2_) and CH_4_ (99.9995%). The gasses were provided by Praxair-España S.L., Spain. The gas flow rates used for SiH_4_, B_2_H_6_ and CH_4_ were in the range of those employed for the preparation of p-a-SiC:H thin films in the complete TFSC devices.

The thickness of the deposited p-a-SiC:H layers was experimentally determined by profilometry measurements on cross-sectioned witness samples using a mechanical step profilometer (Model Ambios XP1, AmbiosTechnology, USA).

The dark conductivity was determined using a p-a-SiC:H single layer on highly resistive glass substrate. The measurements were carried out by using a Four Probe Station designed by SIGNATONE (USA) coupled to a Keithley 4200CS+2SMU+2SMU-PA and a temperature controller (SIGNATONE, Model S-1060R).

### 2.2. UV-NIR Spectrophotometry and Ellipsometry

UV-NIR spectrophotometric measurements were performed with an optic fiber AVANTES, model AvaSpec2048-USB2 (Eerbeek, Holland), able to obtain reflectance and transmittance (R and T) spectra in a wavelength range between 200 nm and 1100 nm. The Brüggeman effective medium approximation (BEMA) and a multilayer model (*Optical* software [22]) were used to fit the R and T spectra measured *ex situ* in the UV-NIR range [23,24]. BEMA approximation is available in the multilayer model in order to implement intermixing of adjacent layers into the optical model [25]. This approximation assumes that the material is composed of an aggregate of small particles, each with its own volume fraction and dielectric constant. In this case, the volume fraction and dielectric constants of a-Si:H and voids were used. Therefore, the fit parameters were the layer thickness and the combination of different volume fractions of a-Si:H and voids. For the best fitting, complex refractive index corresponding to each p-a-SiC:H layers under study were obtained and, consequently, the layer absorption coefficient.

The ellipsometric measurements were carried out by using a variable angle spectroscopic ellipsometer SOPRA, model GES E5 in the energy range 1.5–4 eV at an incidence angle of 74° with a microspot configuration.

### 2.3. Glow Discharge—Optical Emission Spectrometry

GD-OES analysis was performed with a JY 5000 RF instrument manufactured by HORIBA Jobin Yvon (Longjumeau Cedex, France). This instrument is equipped with an rf generator, a standard HJY GD source with an anode of 4 mm internal diameter, two optical spectrometers (a monochromator and a polychromator), and with the Quantum XP software.

High-purity Ar (99.999% minimum purity) from Air Liquide (Oviedo, Spain) was employed as discharge gas. One of the spectrometers consists of a 0.5 m Paschen Runge polychromator (110–800 nm of wavelength range, and a concave grating of 2400 lines mm^−1^) with the optical path purged with nitrogen. The system is also equipped with a Czerny–Turner monochromator (0.64 m focal length, and a planar holographic grating of 2400 lines mm^−1^) which allows the increase of instrument’s capabilities and, therefore, the detection of emissions at any desired wavelength within its spectral range (200–800 nm). Further details of the GD-OES instrument are described elsewhere [26,27]. The emission lines selected in this study (corresponding all of them to atomic transitions) were 121.57 nm for H, 156.14 nm for C, 249.77 nm for B (measured with the monochromator), 288.16 nm for Si and 334.50 for Zn. The voltage applied to the photomultiplier tubes (PMT) was optimized for each wavelength of interest to obtain maximum sensitivity and, finally, PMT voltages were fixed at 910 V for B and 999 V for the other elements.

The operational method “constant pressure-constant forward power” was used throughout the experiments. Experimental conditions (450 Pa Ar discharge pressure and 25 W rf forward power) were chosen as a compromise between high sensitivity and good depth resolution through the analysis of p-a-SiC:H thin films. The shape and depth of the craters produced in the samples after GD-OES analysis were measured by using a mechanical profilometer (Ambios Technology, USA).

## 3. Results and Discussion

### 3.1. UV-NIR Spectrophotometry and Ellipsometry Measurements

**Figure 1.**
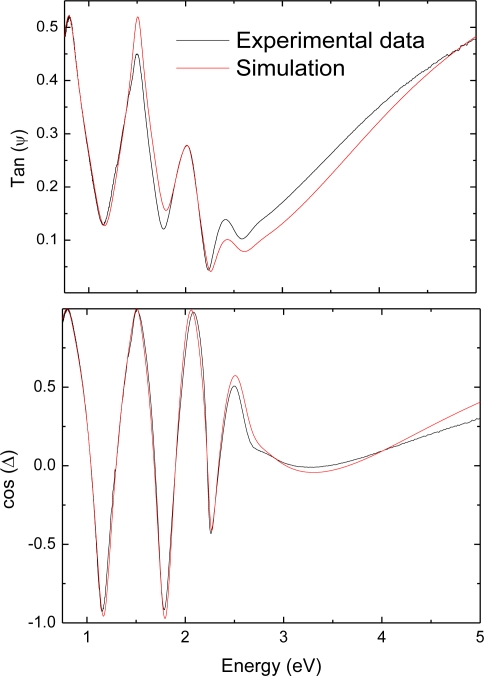
Ellipsometry spectra obtained for sample p-4 (6 sccm SiH_4_, 10 scmm CH_4_ and 20 sccm B_2_H_6_) and corresponding simulations for tan(*ψ*) and cos(Δ).

Figure 1 shows the experimental ellipsometry spectra for one of the samples studied (sample p-4) together with the corresponding simulations for both tan(*ψ*) and cos(Δ). The ellipsometry spectra show large oscillations in cos(Δ), covering almost the full range between −1 and +1, indicating that the angle of incidence was set correctly. The model system used to fit the experimental results consisted of a single layer on a bulk glass substrate or on a Zn substrate, depending on each particular case. The optical constants of the substrates were first determined from measurements on bulk materials with no thin films deposited on them and, thus, they were fixed during the fitting procedure. This way, the unknowns left are the optical constants of the amorphous silicon layers and the layer thickness. Concerning the thickness of the a-Si layers, they were initially set to the values determined by profilometry and further refined during the fitting procedure. On the other hand, in order to determine the optical constants of the layers, the ellipsometric data were further fitted to a Tauc-Lorentz model, including one optical transition. The Tauc-Lorentz model is particularly suited for the analysis of amorphous semiconductors [28] and, in our case, it allows to obtain both the thickness of the p-a-SiC:H thin film and the complex refractive index in the whole studied range. For simplicity, no interdiffusion, surface roughness, *etc.* were considered in the model. This simple model has shown to be adequate to reproduce the experimental features in the full energy range analyzed, leading in all cases to R^2^ values over 0.96. Once the imaginary part of the refractive index has been obtained, it is possible to plot (αE)^1/2^ *versus* the photon energy, where α = 4πk/λ is the absorption coefficient. According to this Tauc analysis [29], the linear extrapolation of (αE)^1/2^ to 0 determines the bandgap. This procedure has led to the data provided in Table 2, where it can be observed that no significant differences were found in the bandgap energy for the four selected samples taking into account the given uncertainties.

The bandgap energy of the p-a-SiC:H thin film was also determined by using a complementary technique: UV-NIR spectrophotometry. For the selected samples, both R and T spectra were obtained in the 200 nm–1100 nm range and the spectra were fitted by using the *Optical* software [22] and BEMA approximation. As was previously explained, the bandgap energy was determined by an iterative process in which both the value of the complex refractive index (combination of a-Si:H and voids volume fractions) and the thickness of the sample are simultaneously modified. It must be noted that the resulting Tauc plot has a different linear regime which denotes the onset of absorption and, therefore, extrapolating this linear region to the abscissa, yields the energy of the optical bandgap of the material. However, if the material of interest does not have a single phase (e.g., for doped amorphous thin film silicon layers), it is possible that it will not have a single distinct absorption onset, which corresponds to a more gradually-sloping curve in the Tauc plot. Thus, a range of bandgap energies, including the maximum and the minimum values obtained, was included in Table 2 for a more precise evaluation of the bandgap energies. As above, once the imaginary part of the refractive index was obtained, Tauc analysis was performed, leading to the different values of the bandgap energy provided in Table 2. In this case, a range of energies was shown for the bandgap energy, being the standard deviation values for three independent measurements in the range of 2–3%. As can be seen in the table, both ellipsometry and spectrophotometry measurements lead to consistent values of bandgap energies for the four selected samples. Additionally, it could be stated that the changes investigated for the gas flow rates in the synthesis stage (SiH_4_ from 6 to 10 sccm and B_2_H_6_ from 12 to 20 sccm) do not produce significant differences in the energy gap of the p-a-SiC:H thin film. However, no information related to dopant elements concentration or their distribution throughout the coating was obtained and, therefore, further analysis should be performed in order to evaluate the influence of the gas flow rates on the doping level achieved, which have a direct effect on the final efficiency of PV devices.

### 3.2. Depth Profiling Analysis of p-a-SiC:H Thin Films by rf-GD-OES

High quality GD analysis of coated samples mostly depends on the depth resolution and, therefore, on the experimental conditions selected for the analysis [30]. As is well known, the crater bottom must be flat within the entire sputtered area (in our case 4 mm) and with the crater walls perpendicular to the sample surface for optimal depth resolution. Working in continuous operation mode, Ar discharge pressure and rf forward power are the experimental parameters to be optimized in order to obtain a good depth resolution as well as maximum sensitivity. The influence of both parameters on the signal intensities and relative depth resolution for the analysis of p-a-SiC:H thin films deposited on Zn substrates has been investigated in a previous work [21], and 450 Pa Ar discharge pressure and 25 W rf forward power were selected as the optimum conditions. Thus, these conditions were selected for all the subsequent measurements.

**Figure 2.**
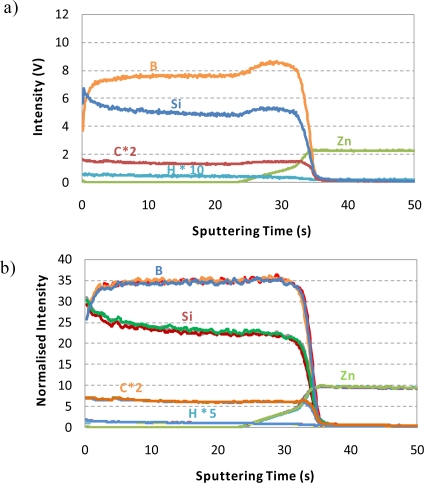
rf-GD-OES depth profiles obtained for sample p-3 (experimental conditions: 450 Pa and 25 W). (**a**) Qualitative depth profile; (**b**) Normalized qualitative depth profiles (with respect to Ar intensity), corresponding to three independent measurements (*i.e*., for each element, three signal profiles are overlapping, showing an excellent reproducibility).

The ability of rf-GD-OES for depth profiling analysis of p-a-SiC:H thin films prepared at different gas flow conditions was first investigated. Figure 2a shows the qualitative depth profile (intensity *versus* time) obtained for sample p-3 and Figure 2b collects the three replicates measured for such sample. In this latter case, normalized intensity signals with respect to Ar intensity were used in order to alleviate possible instrumental instabilities (e.g., signal drift). As can be seen in Figure 2a, a good depth resolution between the p-a-SiC:H layer and the Zn substrate was observed (see the rather vertical shape of B, Si and C signals at the p-layer/Zn interface). Moreover, it can be highlighted that the doping elements of the layer (B, C and H) were perfectly identified in the profile. In this figure, net intensity signals (not normalized) were shown and a small increase of B and Si intensities can be observed at the interface zone, which could be attributed to the presence of Zn from the substrate. The Zn substrate is more conductive than p-layer and, therefore, an increase in the sputtering rate is produced at the interface, growing at the same time the Si, B and C intensity signals. In Figure 2b, normalized intensities are collected, showing a more homogeneous Si and B profiles at the interface zone. Additionally, it should be stressed that the qualitative depth profiles obtained for three independent analyses by rf-GD-OES showed an excellent reproducibility since a perfect overlapping between the intensity signals was obtained for all the elements, both in the p-layer and the Zn substrate.

**Figure 3.**
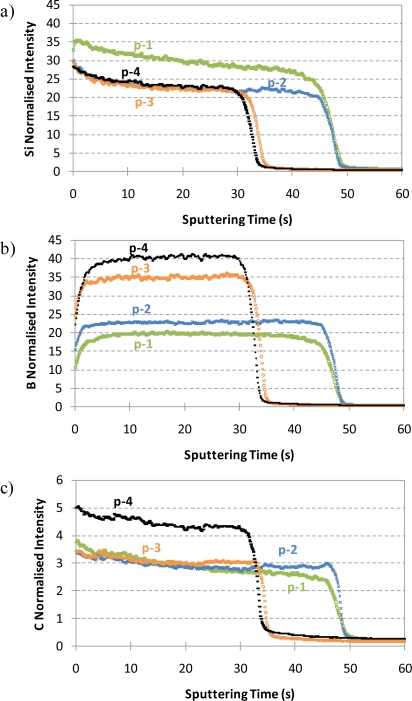
Qualitative depth profiles obtained by rf-GD-OES for samples p-1, p-2, p-3 and p-4 (experimental conditions: 450 Pa and 25 W). In all cases, normalized intensity signals with respect to Ar intensity were used. (**a**) Si normalized signal intensities; (**b**) B normalized signal intensities; (**c**) C normalized signal intensities.

Next, a critical comparison of qualitative depth profiles obtained by rf-GD-OES for the four selected samples (prepared by using different SiH_4_ and B_2_H_6_ flow rates) was carried out. First of all, it should be clarified that, concerning the p-layer deposition process, an increase of the B_2_H_6_ flux reduces the growth velocity rate of the layer, whereas an increase of the SiH_4_ flux produces an increase of the growth velocity rate. Thus, the higher the B_2_H_6_ flow rate, the thinner the p-layer deposited on the Zn substrate and the higher the SiH_4_ flow rate, the wider the p-layer. The deposition time used for the preparation of the p-a-SiC:H thin films was always constant (30 minutes) and the thickness of the layers was experimentally determined by profilometry measurements on cross-sectioned witness samples. Two different thicknesses range were observed for the samples; samples p-3 and p-4 showed a thickness around 420 nm (20 sccm B_2_H_6_), whereas samples p-1 and p-2 showed a higher thickness in the range of 580–600 nm (12 sccm B_2_H_6_). Figure 3 shows the qualitative depth profiles obtained for the four p-a-SiC:H thin films using rf-GD-OES: (a) silicon signal intensities, (b) boron signal intensities, and (c) carbon signal intensities. Although similar bandgap energies were obtained for the four p-layers with the gas flow rates investigated by UV-NIR spectrophotometry and ellipsometry, significant differences were observed in the qualitative depth profiles obtained by rf-GD-OES. Figure 3a shows the depth profiles obtained for Si and, as can be seen, the sputtering time necessary to reach the interface for samples p-1 and p-2 was around 48 s, whereas only 34 s was necessary for samples p-3 and p-4. The sputtering time necessary to reach the p-layer/Zn interface for samples p-1 and p-2 was longer than for samples p-3 and p-4, not only due to the SiH_4_ and B_2_H_6_ fluxes employed, but also because the higher the B concentration in the layer, the higher the conductivity and, therefore, the faster the sputtering time.

In order to estimate the dopant elements levels in the p-layers, the ratio between the area of the Si, B and C signals obtained from the qualitative profiles and the thickness of the layers obtained by mechanical profilometry was calculated. Table 3 collects the estimated levels for Si, B and C in the four p-layers, which directly depend on the different gas flow rates employed (Table 1). Please note that in all cases the thicknesses of p-layers calculated by profilometry were in agreement with those obtained by ellipsometry measurements (see Table 4). Moreover, the relatively large standard deviations obtained for the thickness measurements could be mainly attributed to the surface roughness of the substrates, in the range of 6–10 nm. Samples p-1 and p-2 only differ on the SiH_4_ flux (10 sccm and 8 sccm, respectively) and, therefore, the quantity of B and C in the p-layer should be higher in sample p-2 since B_2_H_6_ and CH_4_ gases are less diluted. A similar trend was also found for samples p-3 and p-4, where B_2_H_6_ and CH_4_ fluxes were kept constant and the SiH_4_ flux varied from 8 sccm to 6 sccm, respectively. As can be seen in Table 3, the B and C estimated levels continuously increased from sample p-1 to p-4. On the other hand, comparing the samples p-1 and p-2 with p-3 and p-4 two different gas flow rates are varying and, therefore, a less direct effect is observed. However, the main difference between the samples is related to the B_2_H_6_ flux, which changes from 12 sccm to 20 sccm and, although samples p-3 and p-4 have a higher dilution factor of the gases, a significant increase of the B estimated level were observed for these samples compared to p-1 and p-2 (up to a 2-fold factor). Therefore, it should be highlighted that rf-GD-OES technique allow us to obtain valuable information of p-a-SiC:H thin films; it is possible to distinguish the dopant elements (B, C and H) as well as to confirm the homogeneous distribution of them throughout the layer. Additionally, significant differences were observed both in the qualitative depth profiles and estimated concentrations for small variations of the gas flow rates (SiH_4_ and B_2_H_6_), which could help us to optimize the preparation stage of p-layers.

### 3.3. Relationship between Conductivity Measurements and Dopant Elements Concentration

The final efficiency of TFSC devices directly depends on the bandgap energy as well as on the conductivity (σ*_d_*) of the p-a-SiC:H layer. To determinate the dark conductivity, the p-a-SiC:H layer need to be deposited on highly resistive glass (such as Corning glass). After that, two coplanar strips of a metal (e.g., Ag) are evaporated, each providing an ohmic contact. This electrical property is determined by using a picoampere meter connected onto coplanar strips of metal evaporated on the p-a-SiC:H layer and applying the following Equation:

$$\sigma_{d}=\frac{I\times w}{V\times l\times d}$$

where *I* is the measured current, *V* is the applied voltage, *d* the thickness of the film, *w* the distance between the metal strips and *l* the length of the strips.

As mentioned above, the main objective of doping a-Si:H with B and C is to modify its electrical conductivity to establish an electrical field necessary for a correct extraction of the electrons generated in the intrinsic film. Therefore, a compromise dopant elements concentration (which can be obtained by varying the gas flow rates) should be employed in the p-a-SiC:H layer in order to get the maximum conductivity of the layer but without increasing the bandgap energy. Although the measurement of bandgap energies in the four selected samples by UV-NIR spectrophotometry and ellipsometry showed similar values for all of them (in the range of 1.8–1.9 eV), significant differences in the B and C concentrations was found by using rf-GD-OES. Therefore, the influence of the dopant elements concentration on the p-layer conductivity was finally investigated.

**Figure 4.**
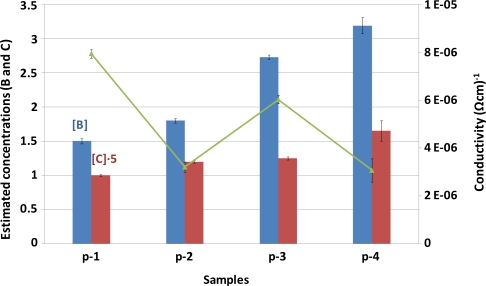
Influence of estimated dopant elements concentrations on the p-a-SiC:H thin films conductivity (samples p-1, p-2, p-3 and p-4). Estimated concentrations correspond to the ratio between the B and C areas and the corresponding layers thickness. Uncertainties values correspond to the standard deviations obtained for three independent measurements.

Figure 4 shows the effect of B and C concentrations on the p-a-SiC:H thin films conductivity (measured in dark conditions and expressed as Ω^−1^cm^−1^). As can be seen, significant changes were observed for the conductivity of p-layer with the different gas flow rates. Although the conductivity of p-layer should proportionally increase with the B concentration, the influence of C concentration was found to be a critical factor: a slight increase on the C concentration results in a noticeable reduction of the conductivity. In this sense, a similar increase of B and C concentrations (17% in both cases between p-1 and p-2 samples) produced a significant decrease of the p-layer conductivity, whereas an increase of 34% in the B concentration (p-2 to p-3 samples) produced a lower improvement. A similar trend to that observed for p-1 and p-2 samples was also found for p-3 and p-4 samples: the raise of C concentration generates a large reduction of the p-layer conductivity.

## 4. Conclusions

Four p-layers, a-Si:H thin films doped with B and C, containing different dopant elements concentration prepared by varying the gas flow rates during the preparation stage were grown on two different substrates: highly resistive glasses and mirror-polished zinc. Glass substrates were used for UV-NIR spectrophotometry, ellipsometry and conductivity measurements whereas conductive substrates were employed for depth profile characterization by rf-GD-OES. Both UV-NIR spectrophotometry and ellipsometry measurements lead to similar values of bandgap energies (in the range of 1.8–1.9 eV) for the four selected samples, suggesting that the changes investigated for the B_2_H_6_ and SiH_4_ flow rates in the synthesis stage do not produce significant differences in the energy gap of the p-a-SiC:H thin film. However, differences were observed in the qualitative depth profiles obtained by rf-GD-OES for the four p-layers, demonstrating the ability of glow discharge sources for a fast and sensitive characterization of TFSC: it was possible to distinguish the different dopant elements in the qualitative depth profiles and to confirm their homogeneous distribution throughout the layer; moreover, the small variations of B_2_H_6_ and SiH_4_ flow rates used in the preparation stage were identified. Additionally, a direct relationship between the p-layer conductivity and the dopant elements concentration was found, *i.e.*, the C concentration being the most critical factor since a slight increase of C level results in a noticeable reduction of the conductivity.

It seems clear that a fast and reliable depth profile characterization of the chemical distribution on a-Si:H TFSC is of critical importance to assist the optimization of the synthesis procedures as well as to evaluate their routine manufacturing quality. Results shown here provide evidence that rf-GD-OES constitutes a promising technique for quality control to ensure optimal performance of p-a-SiC:H thin films and, therefore, to obtain the maximum efficiency of PV devices. Additionally, the present studies are of great interest because they pave the way for further investigations addressed to better understand and control the effect of the contact layers of the a-Si:H film (n-a-Si:H layer/intrinsic a-Si:H layer/p-a-SiC:H layer), interdiffusion processes, *etc*., on the performance of thin film solar cells. The obtained promising qualitative results obtained with rf-GD-OES, call for further investigations to develop proper depth quantification methodologies (to discover both layer composition and possible concentration gradients of non-matrix elements) which will be of great practical importance for the evaluation of final energy conversion efficiency of TFSC, and for quality control of photovoltaic devices production at an industrial level. Moreover, the information obtained by rf-GD-OES from p-a-SiC:H layers profiles, complements that provided by classical electrical measurements and allows a more complete characterization of such solar cell devices. Thus, it is probable that the photovoltaic industry could find an application niche for the fast and reliable technique investigated here.

## Figures and Tables

**Table 1.** Gas flow rates used in the p-a-SiC:H deposition processes. The fabrication of the samples was carried out by using an rf-PECVD cluster system.

**Sample**	**SiH_4_(sccm)**	**CH_4_(sccm)**	**B_2_H_6_(sccm)**	**Total Flow Rate (sccm)**
**p-1**	10	10	12	32
**p-2**	8	10	12	30
**p-3**	8	10	20	38
**p-4**	6	10	20	36

**Table 2.** Bandgap energy for the p-a-SiC:H thin films determined by ellipsometry and UV-NIR spectrophotometry analysis (p-layers deposited on glass substrate).

**Sample**	**Bandgap Energy by Ellipsometry/Tauc (eV)**	**Bandgap Energy by Spectrophotometry^#^(eV)**
**p-1**	1.77 ± 0.04	1.82–1.91
**p-2**	1.83 ± 0.03	1.78–1.85
**p-3**	1.82 ± 0.03	1.81–1.88
**p-4**	1.83 ± 0.03	1.83–1.90

# The standard deviation values for three independent measurement were found to be in the range of 2–3%.

**Table 3.** Si, B and C estimated levels of p-a-SiC:H thin films deposited on Zn. Estimated concentrations correspond to the ratio between the Si, B and C areas and the corresponding layers thickness. Uncertainties values correspond to the standard deviations obtained for three independent measurements.

**Sample**	**Estimated (Si)**	**Estimated (B)**	**Estimated (C)**
**p-1**	2.03 ± 0.05	1.50 ± 0.04	0.20 ± 0.003
**p-2**	1.88 ± 0.06	1.80 ± 0.03	0.24 ± 0.004
**p-3**	1.86 ± 0.03	2.73 ± 0.03	0.25 ± 0.005
**p-4**	1.75 ± 0.03	3.19 ± 0.12	0.33 ± 0.03

**Table 4.** Thickness of the p-a-SiC:H thin film deposited on Zn determined by ellipsometry and mechanical profilometry. Uncertainties values correspond to the standard deviations obtained for three independent measurements.

**Sample**	**Thickness (nm) by Ellipsometry**	**Thickness (nm) by Profilometry**
**p-1**	619 ± 10	606 ± 12
**p-2**	607 ± 10	589 ± 14
**p-3**	448 ± 6	426 ± 18
**p-4**	435 ± 7	414 ± 15
